# On the rules of engagement for microRNAs targeting protein coding regions

**DOI:** 10.1093/nar/gkad645

**Published:** 2023-07-31

**Authors:** Sunil Sapkota, Katherine A Pillman, B Kate Dredge, Dawei Liu, Julie M Bracken, Saba Ataei Kachooei, Bradley Chereda, Philip A Gregory, Cameron P Bracken, Gregory J Goodall

**Affiliations:** Centre for Cancer Biology, an alliance of SA Pathology and University of South Australia, Adelaide, SA 5000, Australia; Centre for Cancer Biology, an alliance of SA Pathology and University of South Australia, Adelaide, SA 5000, Australia; ACRF Cancer Genomics Facility, Centre for Cancer Biology, SA Pathology, Adelaide, SA 5000, Australia; Centre for Cancer Biology, an alliance of SA Pathology and University of South Australia, Adelaide, SA 5000, Australia; Centre for Cancer Biology, an alliance of SA Pathology and University of South Australia, Adelaide, SA 5000, Australia; Centre for Cancer Biology, an alliance of SA Pathology and University of South Australia, Adelaide, SA 5000, Australia; Centre for Cancer Biology, an alliance of SA Pathology and University of South Australia, Adelaide, SA 5000, Australia; Centre for Cancer Biology, an alliance of SA Pathology and University of South Australia, Adelaide, SA 5000, Australia; Centre for Cancer Biology, an alliance of SA Pathology and University of South Australia, Adelaide, SA 5000, Australia; Faculty of Health and Medical Sciences, The University of Adelaide, Adelaide, SA 5000, Australia; Centre for Cancer Biology, an alliance of SA Pathology and University of South Australia, Adelaide, SA 5000, Australia; Faculty of Health and Medical Sciences, The University of Adelaide, Adelaide, SA 5000, Australia; School of Biological Sciences, Faculty of Sciences, Engineering and Technology, The University of Adelaide, Adelaide, SA 5000, Adelaide; Centre for Cancer Biology, an alliance of SA Pathology and University of South Australia, Adelaide, SA 5000, Australia; Faculty of Health and Medical Sciences, The University of Adelaide, Adelaide, SA 5000, Australia; School of Biological Sciences, Faculty of Sciences, Engineering and Technology, The University of Adelaide, Adelaide, SA 5000, Adelaide

## Abstract

MiRNAs post-transcriptionally repress gene expression by binding to mRNA 3′UTRs, but the extent to which they act through protein coding regions (CDS regions) is less well established. MiRNA interaction studies show a substantial proportion of binding occurs in CDS regions, however sequencing studies show much weaker effects on mRNA levels than from 3′UTR interactions, presumably due to competition from the translating ribosome. Consequently, most target prediction algorithms consider only 3′UTR interactions. However, the consequences of CDS interactions may have been underestimated, with the reporting of a novel mode of miRNA-CDS interaction requiring base pairing of the miRNA 3′ end, but not the canonical seed site, leading to repression of translation with little effect on mRNA turnover. Using extensive reporter, western blotting and bioinformatic analyses, we confirm that miRNAs can indeed suppress genes through CDS-interaction in special circumstances. However, in contrast to that previously reported, we find repression requires extensive base-pairing, including of the canonical seed, but does not strictly require base pairing of the 3′ miRNA terminus and is mediated through reducing mRNA levels. We conclude that suppression of endogenous genes can occur through miRNAs binding to CDS, but the requirement for extensive base-pairing likely limits the regulatory impacts to modest effects on a small subset of targets.

## Graphical Abstract

**Graphical Abstract:**
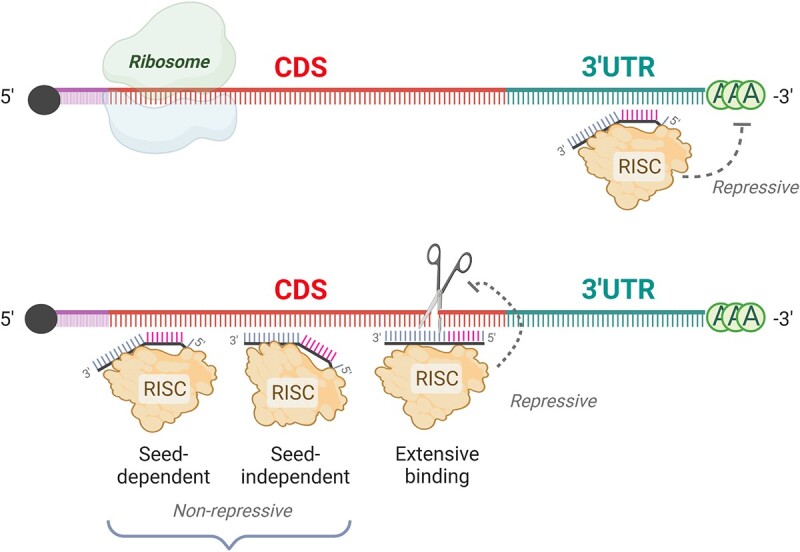

## INTRODUCTION

MicroRNAs (miRNAs) are small non-coding RNAs that constitute the target recognition component of the RNA-induced silencing complex (RISC). In this role, they facilitate gene repression through their recruitment of RISC to their target mRNA transcripts, resulting in translational inhibition or destabilisation of the target mRNA (1). The canonical mechanism through which miRNAs work is well established. This involves the binding of a miRNA to an Argonaute (AGO) protein in such a way as to make a region of the miRNA (nucleotides 2–8 known as the ‘seed’) accessible for complementary base pairing with target transcripts. Initial interactions with the seed then bring about conformational changes in the AGO protein, exposing additional sites within the miRNA (especially nucleotides 12–17) for interaction that can further stabilize binding and facilitate more effective target repression (2–6). In the rare instances of extensive sequence complementarity between the miRNA and its target, or in the case of short interfering RNAs (siRNAs), the interaction across the whole binding interface activates the enzymatic function of AGO2, directly cleaving the transcript at the bases bound to the central region of the miRNA (7–11).

Efforts to understand miRNA-mediated repression initially analysed the effects of miRNA perturbation on the transcriptome. Such studies revealed ‘rules’ of functional sites (also known as microRNA response elements, MREs). For example, longer and fully complementary seed interactions (across 8 contiguous nucleotides) are most effective (12–14). Also, MREs located within 3′UTRs are more effective than sites within protein coding sequences (CDS) (12,13,15,16), presumably due to avoidance of competition from transiting ribosomes (15,17). These observations have been widely replicated and have set the landscape for miRNA target prediction to such an extent that potential sites within coding exons are often entirely ignored (12,18–26).

Despite this, individual examples of functional miRNA targeting within CDS regions have been reported (27–29). AGO cross-linking and immunoprecipitation studies reveal extensive miRNA interaction sites within coding regions, with the frequency of binding to the CDS often similar to that within the 3′UTR (30). Of particular interest is the report that miRNAs can target the CDS via a mechanism that is independent of the seed, but that is dependent upon extensive binding elsewhere, particularly at the 3′ end of the interaction site (31). The mechanism of gene repression for these CDS sites was reported to occur through aborted translation and to not affect the level of the target transcript. Such interactions are therefore likely to have been missed in most studies because typically only the effects on mRNA levels are examined. If such a mechanism is substantiated, the breadth of functional CDS targeting by miRNAs may be vastly larger than previously recognised.

In this study, we have sought to definitively determine the capacity of miRNAs to exert their repressive effects within protein coding regions. Using multiple reporter constructs, miRNAs and cell lines, we find that miRNAs are capable of repressing their targets within protein coding regions, however in contrast to observations from the Zhang et al. study (31), the mode of target repression is canonical (seed-dependent), is not especially dependent on the binding of the miRNA 3′ terminus and operates at the level of transcript stability. We find that an extensive binding interface between the miRNA and its target is required for functionality within the CDS, which involves direct cleavage of the target transcript. Bioinformatic assessment of both predicted and experimentally identified binding sites reveal that CDS targeting is likely to occur endogenously, but the requirement for extensive base pairing will limit this mechanism to a relatively small number of genes.

## MATERIALS AND METHODS

### Cell culture and antibodies

MDA-MB-231, MCF7 and HEK293T cells were cultured in DMEM supplemented with 10% FBS. PC3 and HeLa cells were cultured in RPMI-1640 supplemented with 10% FBS. Cells were subcultured every 2–3 days using 0.2% Trypsin/1× PBS to dissociate cells from the plate during passaging. All cell lines tested negative for mycoplasma. The antibodies used are as follows: anti-Acin1 (Rabbit pAb; Bethyl Laboratories; Cat#BETHA300-999-AM), anti-Bard1 (E11, mouse mAb; Santa Cruz; Cat# SANTSSC-74559); anti-CDH2 (Rabbit mAb; Cell Signalling; Cat# 13116), anti-CTNNB1 (mouse mAb; BD Biosciences; Cat# 610154), anti-KRT5 (E2T4B, Rabbit mAb; Cell Signalling; Cat# 71536T), anti-MAP2K1 (E342, Rabbit mAb; Abcam; Cat# ab32091), anti-Met (Met (D1C2) Rabbit mAb; Cell Signalling; Cat# 8198), anti-NOTCH2 (D76A6, Rabbit mAb; Cell Signalling; Cat# 5732T), anti-RTN4 (Nogo (C-4) Mouse mAb; Santa Cruz Biotechnology; Cat# SANTSSC-271878), anti-Tubulin (Tubulin Mouse mAb; Abcam; Cat# ab7291), Anti-mouse IRDye 800CW (Goat; Li-Cor; Cat# 926–32210), Anti-rabbit IRDye 800CW (Goat; Li-Cor; Cat# 926-32211), Anti-mouse IRDye 680RD (Goat; Li-Cor; Cat# 926–68070) and Anti-rabbit IRDye 680RD (Goat; Li-Cor, Cat# 926–68071).

### Luciferase reporter constructs

The psiCHECK-2 reporter vector (Promega) was initially digested with NruI and NotI, into which were cloned double stranded DNA oligonucleotides (G-blocks, IDT) to introduce an AgeI restriction site 6 nucleotides upstream of the stop codon. The modified psiCHECK-2 plasmid was then digested with AgeI and NotI to insert the desired miRNA target sequences by T4PNK (NEB) treatment and annealing of single stranded oligonucleotides. XhoI and NotI sites present in the original Renilla luciferase vector were used for the cloning of miRNA binding sites into the 3′ UTR. Sequences of G-blocks and single stranded oligonucleotides are listed in Supplemental Table S4.

### Plasmid transfection and dual luciferase assay

Cells were seeded at 5 × 10^4^ cells per well in 24-well plates and transfected the next day with 5 ng psiCHECK-2 or modified psiCHECK-2 along with either 5 nM of control or miRNA mimic or 20 nM of control or miRNA inhibitor diluted in opti-MEM (Invitrogen) and in combination with lipofectamine-2000 (Invitrogen). The transfection reagent was replaced with fresh cell growth media 6 hours post transfection. Luciferase activity was measured 48 h post transfection using a dual luciferase kit (Promega).

### RNA isolation, cDNA synthesis and qRT-PCR

Cells were seeded at 8 × 10^4^ cells per well in 6-well plates. The following day, cells were transfected with 10 nM miRNA mimic or 50 nM miRNA-inhibitor diluted in opti-MEM (Invitrogen) and in combination with Lipofectamine RNAiMAX (Invitrogen) using the recommended protocol. Media was replaced 6 h post transfection. 72 hours post transfection, total RNA was harvested using TRIzol (Invitrogen), following the standard manufacturer's protocol. cDNA was synthesised using the QuantiTech Reverse Transcription Kit (Qiagen) from 1 μg of RNA. qRT-PCR was performed on a Rotor-Gene 6000 series thermocycler (Qiagen) with Master SYBR Green reagent (Qiagen). Analysis was performed using the comparative quantitation feature in the Rotor-Gene software with each gene measured being normalized to the mean of GAPDH and RPL32. All miRNA-mimics, miRNA-inhibitors, transfected pseudomiRs and qPCR primers are listed in Supplemental Table S4.

### Protein purification and western blotting

Cells were seeded at 8 × 10^5^ per well in 6 well plates. The following day, cells were transfected with 10 nM miRNA mimic using Lipofectamine RNAiMAX (Invitrogen) using the recommended protocol. The transfection reagent was removed 6 hours post transfection and cells were cultured in fresh media. Seventy-two hours post transfection, cells were treated with ice cold 1× RIPA lysis buffer prepared by the recommended combination of cOmplete Mini, EDTA-free Protease-inhibitor Cocktail tablet (PIC; Roche), PhoSTOP EASYpack (Roche), 10× RIPA buffer (Abcam). The concentration of protein in purified lysate was estimated using Pierce BCA Protein Assay Kit (Thermo Scientific). 20 μg of protein was loaded onto Bolt Bis–Tris Plus gels (gel type based on protein size) using 1× Bolt MOPS SDS Running Buffer (Invitrogen). Proteins were transferred to nitrocellulose membrane at 4^o^C using 1× Bolt Transfer Buffer (Invitrogen) with 10% methanol by volume. Membranes were incubated with Ponceau stain for total protein visualization using ChemidocTouch. Membranes were blocked in 5% skim milk for 1 h at room temperature and incubated overnight in the recommended dilution (generally 1:1000) of primary antibody at 4°C. Protein visualization using Near Infrared (NIR) was achieved by incubation for an hour at 4°C in secondary (1:20 000; PBST) antibody, IRDye 800, of the correct species. The same membrane was re-probed with α-tubulin (1:2500 dilution) for an hour at 4°C followed by an hour of secondary (1:20 000; PBST) antibody, IRDye 680.

### Lentivirus production and infection

For lentivirus production, HEK293T cells were plated at 2 × 10^6^ cells in T25 flasks. The following day, cells were transfected with 1 μg pLP1, 1 μg pLP2, 1 μg pLP-VSVG, 1 μg pTAT and either 4 μg of the pLV4301-enhanced GFP transfer vector (32) or 4 μg of the pLX301-mCherry transfer vector (33). DNA was mixed in 500 μl opti-MEM and transfected in combination with 12 μl Lipofectamine-2000. Transfection reagent was removed 6 h post transfection and viral supernatant of either pLV4301-eGFP or pLX301-mCherry was collected after 72 hours. MDA-MB-231 cells were seeded at 2 × 10^6^ cells in T_25_ flasks. The following day, cells were transduced (1:4) with viral supernatant of pLV4301-eGFP in the presence of polybrene (4 mg/ml). MDA-MB-231-eGFP positive cells were re-transduced with pLX301-mCherry viral supernatant to generate a pool of MDA-MB-231-eGFP + mCherry cells. The transduced pool of cells was selected using puromycin (1 μg/ml) and grown for at least 48 h before further analysis.

### Single cell sorting and flow cytometry

MDA-MB-231-eGFP-mCherry cells were washed twice with warm washing buffer (1× PBS + 10 mM EDTA) followed by short incubation with 3 mM EDTA in 1× PBS. Semi-detached cells were treated with TrypLE followed by dilution in 1× PBS + 10 mM EDTA. Cells were centrifuged at 350×g for 5 min, washed with 1× PBS + 10 mM EDTA and resuspended in sorting buffer (ice cold 1× PBS, 5 mM EDTA, 1% FCS and 25 mM HEPES; pH 7.0). Cells were then filtered using a 30 μm Filcon sterile filter (BD Biosciences) and sorted on the basis of fluorescence intensity compared to control cells including parental MDA-MB-231 (no colour), MDA-MB-231-eGFP (single colour) and MDA-MB-231-mCherry (single colour). Single cells separated in 96-well plates were grown in the conditioned media before transferring into larger 6-well plates. Flow Cytometry sorting was performed on the MoFlo Astrios EQ High Speed Cell Sorter using Summit Software version: 6.3.1 (Beckman Coulter, Miami, FL, USA). Experiments utilised the 488 nm (150 mW) and 561 (200 mW) laser lines and the 100 micron nozzle at 30 PSI. Laser and light filter usage are displayed on plot axes. No forward scatter masks were used. Flow cytometry data was analysed using the Apple Macintosh-version of FCS express 6 (De Novo Software, Los Angeles, CA, USA).

### Primer extension assays

15 pmoles of primers P1 and P2 were 5′ end-labelled with equimolar amounts of ^32^P- γ -ATP using T4-PNK and purified through G-25 columns (GE Healthcare 27-5325-01). The Rps12 control primer was similarly labelled using a 2:1 ratio of cold:hot ATP. 10ug of total RNA extracted MDA-MB-231-eGFP-mCherry cells transfected with pseudo-miRs was mixed with 0.5 pmole each Rps12 and P1 or P2 ^32^P-labeled primers, denatured at 75°C for 5 min then reverse transcribed using Superscript III (Invitrogen) according to the manufacturer's instructions. Sanger DNA sequencing of the mCherry reporter with the same radiolabelled primers and Klenow DNA polymerase (NEB M0210) was used as a ladder to map the cleavage sites to nucleotide resolution. Products were separated by large format 5% acrylamide, 7M urea PAGE, exposed to a phosphor screen and imaged using a Typhoon. Primer sequences were mCherry P1: TTGACCTCAGCGTCGTAGTG, P2:TACTTGTACAGCTCGTCCATG, Rps12: GCAGTCTTCAGAACCTCTTG.

### Statistical analysis

Each experiment was performed across multiple biological replicates as indicated in the individual figure legends. Data are presented as mean ± s.e.m., and *P* values were determined by two-tailed Student's *t* test.

### Bioinformatic prediction of miRNA binding

For the prediction of miRNA binding shown in Supplemental Table S1, analysis was restricted to a subset of 560 miRNAs (either annotated as ‘high confidence’ in miRBase (34) and expressed ≥10 rpm or annotated as ‘low confidence’ and expressed at ≥1000 rpm). Sequences (3′UTR and CDS) were extracted from ENSEMBL Biomart (35).

### Bioinformatic analysis of AGO-CLASH

Processed CLEAR-CLIP data from mouse keratinocytes was obtained as supplemental files from GEO (Accession GSE102716 (36)) which comprised, for each read, the mapped location of the target RNA part of the read and the name of associated microRNA. Analyses were performed using the *Mus musculus* genome version ‘mm10’ and UCSC gene transcripts in python using pyreference (https://pypi.org/project/pyreference/), HTSeq (37) and seaborn (38) libraries. Using ‘Set1 Control’ sample file (161692 CLEAR-CLIP reads) and all the UCSC transcripts, we annotated each read target region as overlapping intronic, exonic, 3′ UTR and/or 5′ UTR gene regions, discarding unannotated (i.e. intergenic) reads. To compensate for nuclease ‘nibbling’, the genomic interval of the target RNAs was expanded on both ends by three bases. The RNAduplex method from the Vienna RNA package (39) was used to analyse the binding affinities of resulting target RNA and miRNA sequences. The resulting dot-bracket annotation and delta-G values for the predicted RNA duplexes and other annotation were used to produce the table of CLEAR-CLIP read annotations provided as Supplemental Table S1. Where present, duplicate reads (reads with identical RNA target intervals and microRNAs) were counted (in the ‘counts’ column) and collapsed into a single entry. For visualisations, reads are classified as in ‘3′ UTR’ if they overlap 3′UTRs and ‘coding region’ if they are in exons and not in 3′ or 5′ UTRs.

## RESULTS

### MiRNAs frequently interact with coding regions

**Figure 1.**
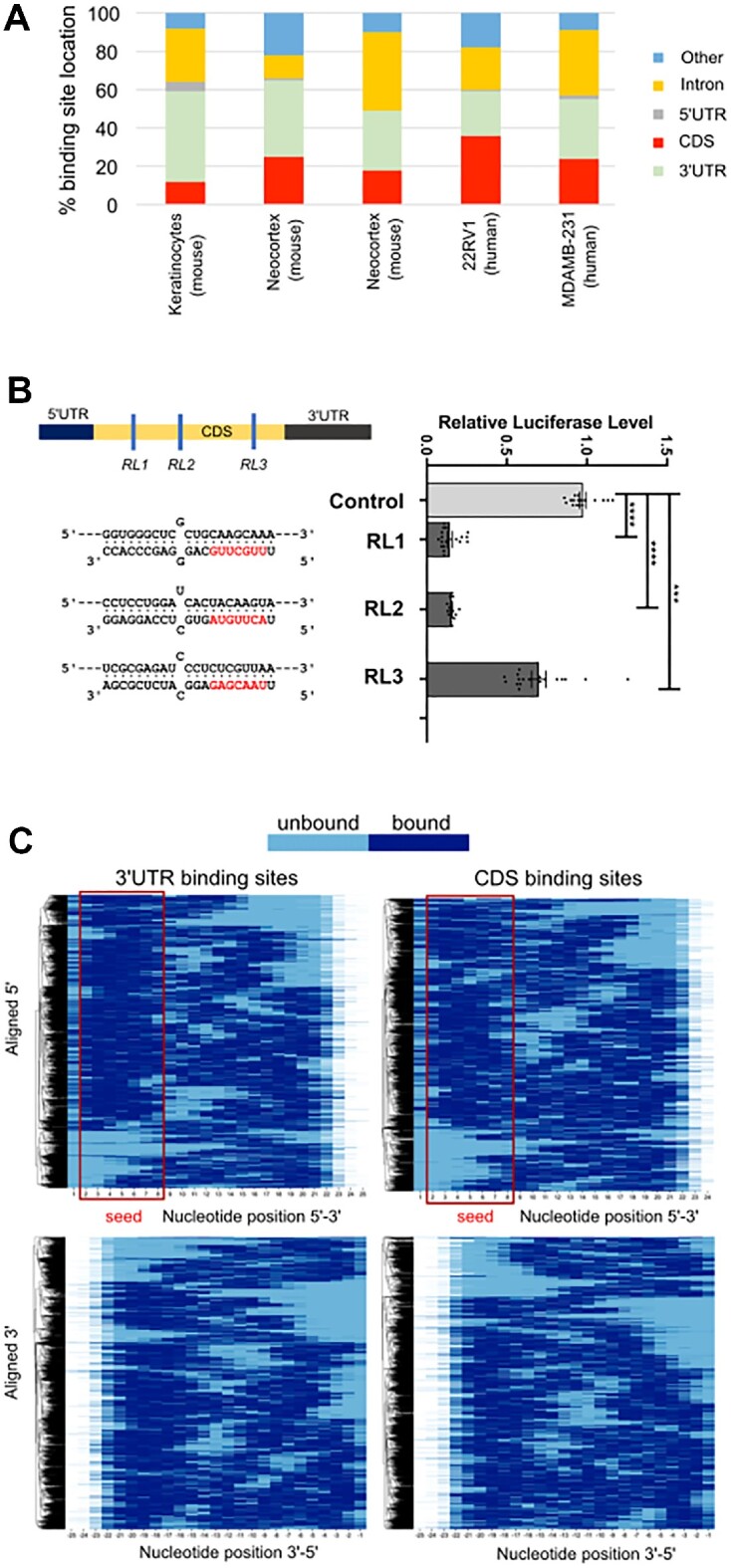
Locations of miRNA interaction within mRNAs and of base pairing within the miRNAs. (**A**) Locations of miRNA interaction as determined by HITS-CLIP. The references of the relevant published study from left to right are as follows (36,40,42,45,46). (**B**) Effects on artificial miRNAs targeting three different regions in the Renilla luciferase reporter gene. The locations of binding within the mRNA are shown schematically and base pairings are shown with the miRNA seed regions in red. Luciferase reporter assays were performed in MDA-MB-231 cells. Quantitative data are based on three biological replicates, with each experiment containing 6 technical replicates. Data is expressed as mean ± s.e.m. Statistical significance (*P < 0.05, **P < 0.01, ***P < 0.001 and ****P < 0.0001) was determined by two-tailed Student's *t* test. (**C**) Patterns of base pairings for miRNAs binding in 3′UTRs (left panels) and CDS regions (right panels) using data from the CLEAR-CLIP study of Hoefert *et al.* (36). Paired bases are in dark blue. Alignments are from the 5′ end of the miRNA in the upper panels and the from the 3′ end of the miRNA in the lower panels.

The Ago-HITS-CLIP procedure identifies the locations of miRNA interaction within their target mRNAs in live cells. Although miRNAs are generally assumed to act through interaction with mRNA 3′UTRs, Ago-HITS-CLIP in a range of different cell types reveals a substantial amount of binding in protein coding sequences (CDSs) (Figure 1A), consistent with reports showing that miRNAs can target CDS regions (27,36,40–42). Furthermore, when we designed artificial miRNAs to target three different regions within the CDS of a Renilla luciferase reporter mRNA, containing in each case a mismatch at position 12 of the miRNA to minimise direct cleavage of the target, we found these miRNAs all substantially reduced activity of the targeted Renilla luciferase relative to activity of the co-expressed but non-targeted firefly luciferase (Figure 1B). One of the artificial miRNAs was less effective than the other two, but this was likely due to the target sequence being within a predicted hairpin structure (Supplemental Figure 1). These data support the contention that miRNAs binding within the CDS can repress expression.

A non-canonical form of binding in CDS regions that requires the 3′ end of the miRNA to be base paired to the target mRNA has been reported (31). To assess how common this mode of binding is in CDS and 3′UTR regions we performed a broad survey of the base pairing interactions of miRNAs by analysing the interactions found by CLEAR-CLIP (covalent ligation of endogenous Argonaute-bound RNAs, also known as cross-linking ligation and sequencing of hybrids (CLASH)), using data from Yi and colleagues (36). The CLEAR-CLIP procedure ligates the miRNA to the fragment of target mRNA to which it is bound, thereby identifying both the miRNA and the target sequence. We compiled the base pairing interactions for CDS and 3′UTRs, and because different miRNAs are not all of identical length, we performed separate alignments with anchoring at the 5′ end of the miRNA (thereby aligning the seed regions) or at the 3′ end to determine whether base pairing of miRNA 3′ ends is especially prominent in CDS interactions. This analysis indicated that base pairing patterns in the CDS are similar to those observed in 3′UTRs and that the non-canonical mode of binding, with base pairing at the miRNA 3′ end, occurs at similar low frequency in CDS and 3′UTRs (Figure 1C).

### Repression by binding to the CDS requires seed complementarity but not 3′ end pairing

**Figure 2.**
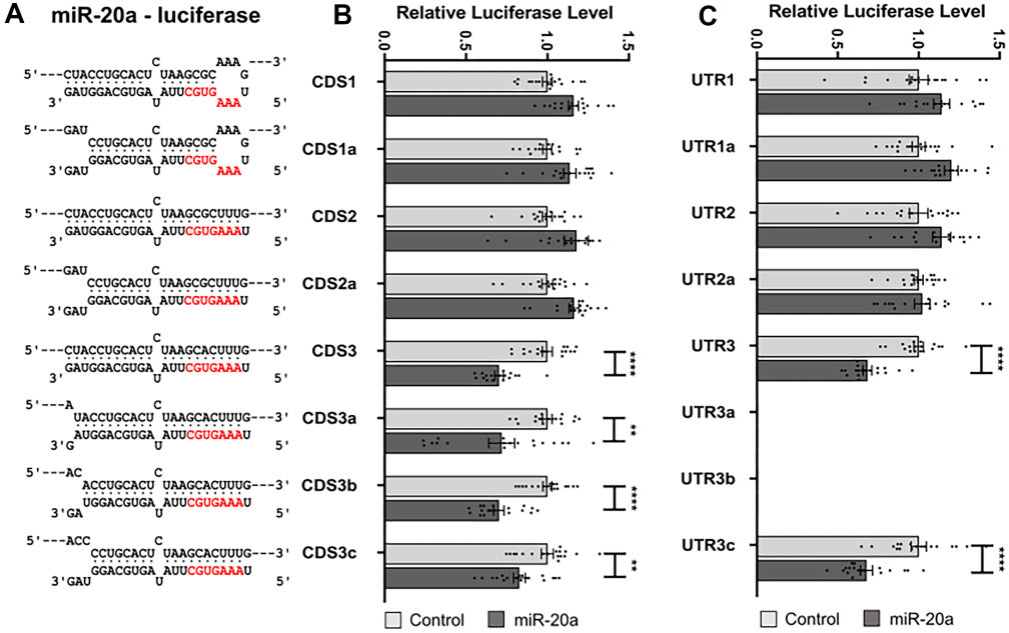
Base-pairing interactions required in the CDS or 3′UTR for miR-20a to effectively target a luciferase reporter gene. (**A**) Binding models indicating base pairing interactions between miR-20a (bottom) and the miRNA-response element (MRE, top) that is cloned within the luciferase CDS or 3′UTR. The miRNA seed region is shown in red. (**B, C**) Results of Renilla Luciferase reporter assays after co-transfection of miR-20a mimic in MDA-MB-231 cells. Data are expressed as mean ± s.e.m., *n* = 18. Statistical significance (*P < 0.05, **P < 0.01, ***P < 0.001 and ****P < 0.0001) was determined by two-tailed Student's *t* test.

Given the frequency of CDS interaction sites indicated by CLIP studies, we sought to further interrogate the base pairing requirements for active repression of expression via binding to CDS regions. We introduced potential binding sites for various endogenous miRNAs into the coding region of Renilla luciferase, using the strategy employed by Zhang *et al.* (31), in which a unique restriction site (encoding two additional amino acid residues) is inserted upstream of the stop codon, allowing subsequent insertion of additional target sequences for selected miRNAs. We first examined whether repression by miR-20a requires seed region and/or 3′ end base pairing. In contrast to the findings of Zhang *et al.*, we found that the criteria for miR-20a repressive effect were similar whether the binding site was in the CDS (Figure 2A, B) or the 3′UTR (Figure 2A, C), with seed region binding being required, but not binding by the miRNA 3′ end. When the seed region was base paired there was effective repression (CDS3, CDS3a, CDS3b and CDS3c in Figure 2), whereas complete base pairing of the miRNA 3′ end (CDS1) did not compensate for seed region mismatches, and even a minor imperfection in the seed region (CDS2 and CDS2a) eliminated repression by the miRNA, regardless of 3′ end base pairing.

**Figure 3.**
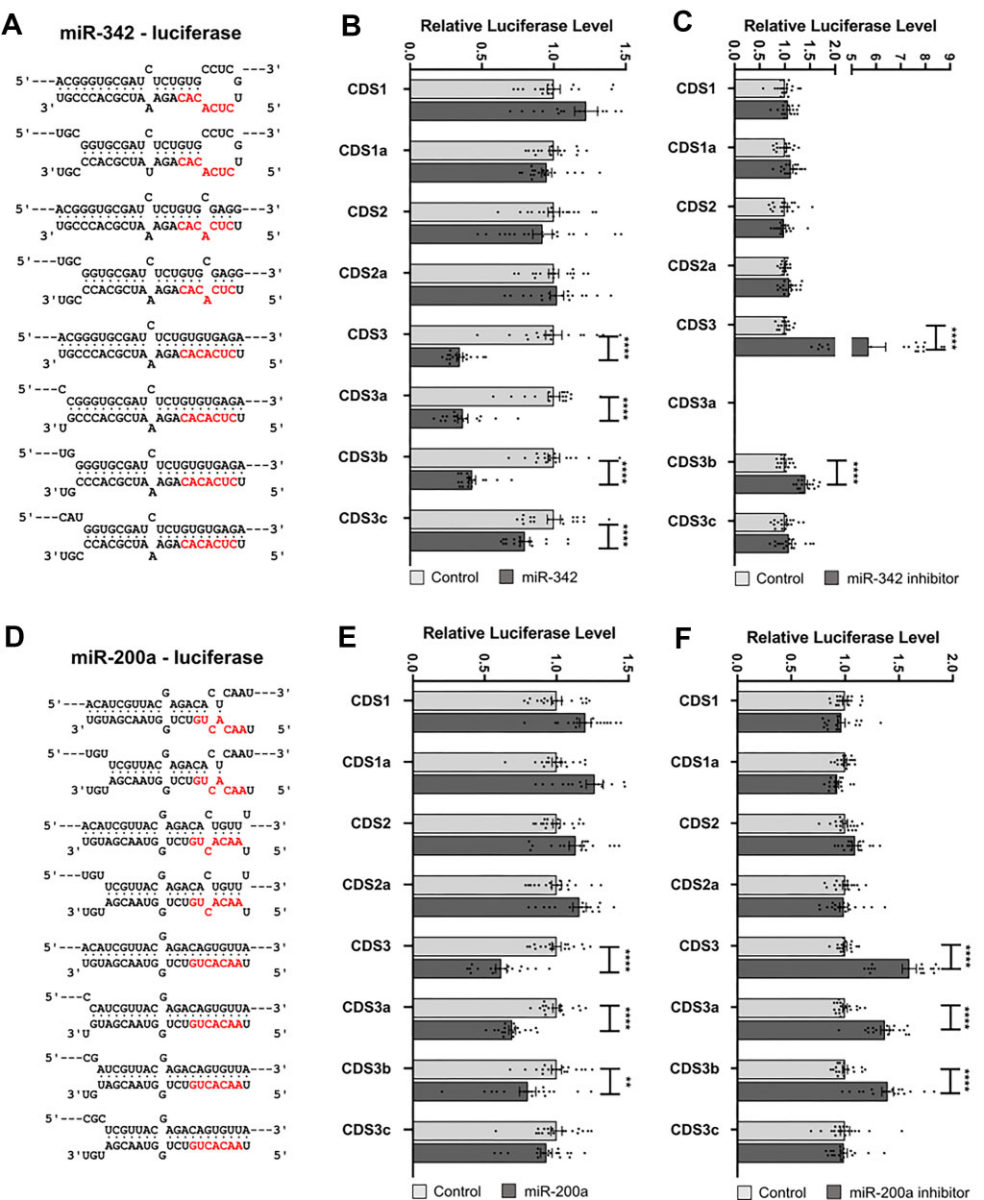
Base-pairing interactions required in the CDS or 3′UTR for miR-342 and miR-200a to effectively target the CDS of a luciferase reporter gene. (**A, D**) Binding models indicating base pairing interactions between the miRNA and the miRNA-response element cloned within the luciferase CDS. The miRNA seed region is shown in red. (**B, E**) Results of Renilla Luciferase reporter assays after co-transfection of (B) miR-342 or (E) miR-200a mimic in MDA-MB-231 cells. (**C, F)** Results of Renilla Luciferase reporter assays after co-transfection of miRNA inhibitors in MCF7 cells. Data are expressed as mean ± s.e.m., *n* = 18. Statistical significance (*P < 0.05, **P < 0.01, ***P < 0.001 and ****P < 0.0001) was determined by two-tailed Student's *t* test.

As there was no rationale why miR-20a would specifically target CDS, we selected additional miRNAs to assess whether the base pairing criteria for repression within the CDS were similar. These miRNAs were selected on the basis that they are well studied, widely expressed and represented in CLASH data. Initially starting with miR-342, we found the criteria for miR-342 targeting within the luciferase CDS were similar to those seen with miR-20a (Figure 3 compared to Figure 2). Disruption of base pairing of the seed region impaired activity (Figure 3A,B), whether the 3′ end was base paired (CDS1, CDS2) or not (CDS1a, CDS2a), while the miRNA inhibited luciferase activity if the seed region was perfectly base paired, whether or not the 3′ end was also base paired (CDS3, CDS3a, CDS3b, CDS3c). To check that these key criteria of functionality applied at physiological levels of miRNA, we measured the effect of inhibition of the endogenous miR-342 in MCF7 cells, a cell line in which miR-342 is naturally expressed. Inhibition of miR-342 did not affect expression of the luciferases with seed region mismatches (Figure 3C; CDS1, CDS1a, CDS2, CDS2a), regardless of the degree of base pairing at the miRNA 3′ end. The miR-342-CDS3 luciferase (which was strongly inhibited by transfected miR-342 in MDA-MB-231 cells) was strongly activated by inhibition of endogenous miR-342 in MCF7 cells, indicating that the anti-miR inhibitor is effective and that endogenous miR-342 targets the reporter, as expected (Figure 3C). The miR-342-CDS3c luciferase, which has seed pairing but not 3′ end pairing, was not activated by miR-342 inhibitor (Figure 3C), despite being inhibited to a degree by miR-342 in transfected MDA-MB-231 cells (Figure 3B), while the CDS3b luciferase, which has a two base mismatch at the miRNA 3′ end, was only slightly activated on inhibition of the miRNA (Figure 3C). Taken together these data indicate that seed region base pairing is essential for inhibition by miR-342, and can be augmented by 3′ end binding, but without a specific requirement for base pairing of the 3′ terminal bases as previously reported (31).

To further check the generality of the base pairing requirements we also tested equivalent luciferase constructs with CDS sites for miR-200a (Figure 3D–F), miR-200b (Supplemental Figure S2A–C) and miR-194 (Supplemental Figure S2D, E). All of these gave similar results, demonstrating that for miRNAs to repress genes via CDS sites, extensive base pairing is required that includes the seed, but does not necessarily include the 3′ terminal nucleotides. We rationalize that if such sites are significant in biology, one would expect similar ‘rules’ to operate across different cells and different miRNAs. However, as we did not find these sites to be functional as was reported previously, we sought to exactly replicate the reporters used in the prior study.

**Figure 4.**
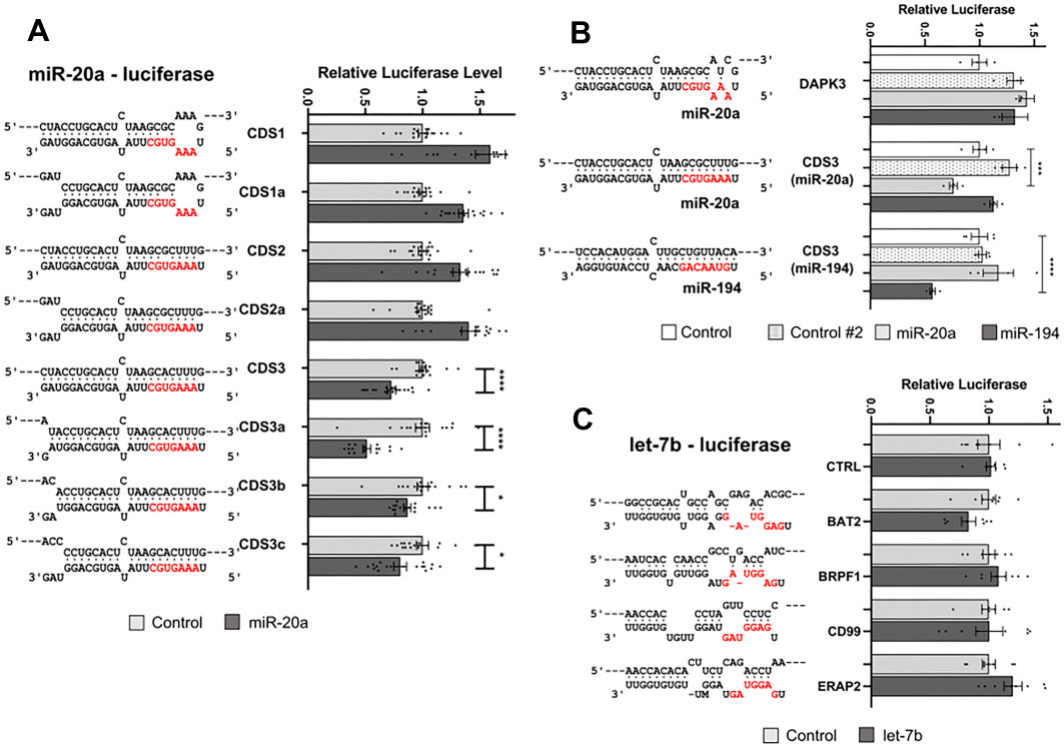
Non-canonical targeting of CDS-luciferase reporter genes is ineffective. (**A–C**) Binding models indicating base pairing interactions between the miRNA and the miRNA-response element cloned within the luciferase CDS are shown. The miRNA seed region is shown in red. Results of Renilla Luciferase reporter assays after co-transfection of (A) miR-20a mimic, (B) miR-20a or miR-194 mimic or (C) let-7b mimic with the reporters indicated. In (B), the DAPK3 reporter is an exact nucleotide match of the reporter used in the Zhang *et al.* (31). The CDS3 constructs are relevant for miR-20a or miR-194 as indicated. In (C), potential let-7b response elements derived from the genes shown again match those reported by Zhang *et al.* All transfections were performed in HeLa cells. Data are expressed as mean ± s.e.m. Statistical significance (*P < 0.05, **P < 0.01, ***P < 0.001 and ****P < 0.0001) was determined by two-tailed Student's *t* test.

One difference between the miR-20a targeting presented in Figure 2 and the Zhang et al. study is the cell line in which the assays were conducted. We therefore repeated our reporter assays in HeLa cells as the prior study had used, but again found no seed-independent CDS repression (Figure 4A). To check whether the difference between our observations and those of Zhang et al. might be due to the seed region mismatch bases in our DAPK3-derived miR-20a reporter being different from those of Zhang *et al.*, we created an additional reporter with identical sequence to that of Zhang et al., but we found it too was not inhibited by miR-20a (DAPK3, Figure 4B). However, restoring base pairing in the seed region resulted in inhibition of the reporter (CDS3, Figure 4B). A reporter with near perfect complementarity to miR-194 was inhibited by miR-194 but not miR-20a. Similarly, miR-20a but not miR-194 inhibited a miR-20a reporter, confirming the specificity of these assays (Figure 4B, Supplemental Figure 3A). To compare further with the Zhang study, four let-7b target reporters were also cloned into the Renilla luciferase CDS that were previously reported to be strongly suppressed upon let-7b transfection (Figure 4C). Again, we report no equivalent finding, though let-7b itself was functional as it effectively repressed a complementary reporter (Supplemental Figure 3B). Collectively, these results all lead to the same conclusions: for miRNA binding in the CDS to be repressive, seed region base pairing is essential, as is extensive base pairing beyond the seed, however specific base pairing of the very 3′ end is not essential.

**Figure 5.**
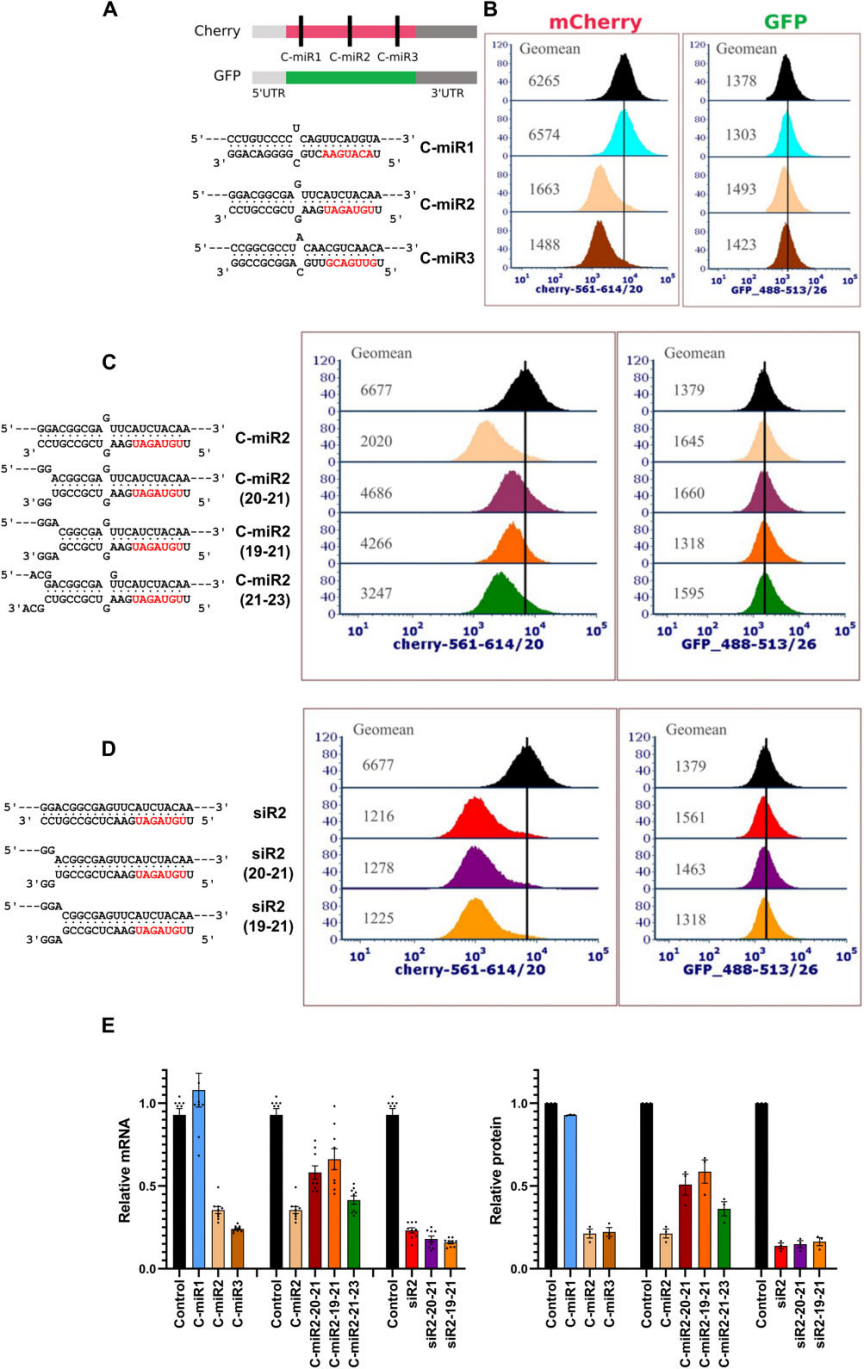
Effects of artificial miRNAs targeting the CDS of an mCherry reporter. (**A**) Predicted base pairing interactions of artificial miRNAs targeting the mCherry CDS in three different locations. (**B**) MDA-MB-231 cells stably expressing mCherry and GFP were transiently transfected with the indicated miRNAs and the mCherry and GFP protein levels quantitated by flow cytometry. The vertical black line is located at the mean fluorescence value of cells transfected with control miR (top) which does not target either reporter gene. Histograms moving to the left indicate repression of the reporter gene (which results in reduced fluorescence in the cell). Mean levels of fluorescence are indicated. (**C**) Effects of disruption of base pairing at the 3′ end of the artificial miRNA. Peak fluorescence values are shown for mCherry transfected with each miRNA. (**D**) Effects of disruption of base pairing at the 3′ end of otherwise perfectly complementary miRNA mimics. (**E**) Relative mCherry mRNA and protein levels in cells transfected with the indicated artificial miRNAs. Data are expressed as mean ± s.e.m., *n* = 3. Statistical significance (*P < 0.05, **P < 0.01, ***P < 0.001 and ****P < 0.0001) was determined by two-tailed Student's *t* test.

To check that the base pairing requirements we identified as being necessary for repression within the CDS were not restricted to the luciferase reporter system, we created a dual colour reporter system with constitutive mCherry and GFP expression in MDA-MB-231 cells and measured the effects of artificial miRNAs targeting the mCherry CDS. An advantage of this system is that the effect of the transfected miRNAs is measured in every individual cell by flow cytometry, giving thousands of data points per transfection for both the targeted mCherry and the non-targeted GFP control. We assessed the effects of miRNA mimics targeting three different regions in the CDS of the mCherry (Figure 5A). As expected, none of these mCherry-targeting miRNA mimics (called C-miRs) affected GFP expression. Two of the miR mimics (C-miR2 and C-miR3) strongly reduced mCherry expression, further demonstrating the potential for highly complementary miRNAs to target CDS regions (Figure 5B).

To assess the role of miRNA 3′ end binding in this reporter context, we disrupted base pairing of the 3′ end of C-miR2. C-miR-20–21 with two bases mismatched at the 3′ end and C-miR2-19-21 with 3 bases mismatched had similar effects, reducing the efficacy of the miRNA but not eliminating miRNA function (Figure 5C). To indicate whether the reduced efficacy was due to reduced binding or was due to the presence of single-stranded bases at the end of the miRNA, we made a longer version of the miRNA that still had 3 unpaired bases at the 3′ end, but retained 8 of the 9 base pairs in the 3′ half of the miRNA (C-miR2-21-23 in Figure 5C). This miRNA was more effective than the shorter C-miR2-19-21, which also has 3 mismatched bases at the 3′ end, indicating that the stability of the duplex is a major criterion for efficacy, rather than the presence or absence of unpaired bases at the 3′ end of the miRNA. Moreover, in the context of complete base pairing of the miRNA across the central region (which allows direct target cleavage by Ago2), the presence or absence of two or three unpaired bases at the 3′ end was also of no consequence (Figure 5D), again indicating that base pairing per se at the 3′ end is not necessary for productive interaction in CDS MREs.

### CDS-targeting miRNAs promote mRNA degradation

To investigate whether the inhibitory effect of the miRNAs targeting the CDS was primarily through mRNA destabilisation or inhibition of translation, we compared the effect of the mCherry-targeting miR mimics on the mCherry mRNA and protein levels. For all of the miR mimics tested, the effect on protein level was closely matched by the effect on mRNA level (Figure 5E). Thus, the predominant effect of the miRNA targeting was on mRNA stability, with little additional effect on translation efficiency. This was the case both when the 3′ end of the miRNA mimic was unpaired (C-miR2-20-12, C-miR2-19-21, C-miR2-21-23, siR2-20-21 and siR2-19-21 in Figure 5E) and when the 3′ end was base paired (C-miR2, C-miR3 and siR2 in Figure 5E), indicating that the translational mechanism of repression by CDS-targeting miRNAs reported by Zhang *et al.* (31) did not have a role in any of these instances.

**Figure 6.**
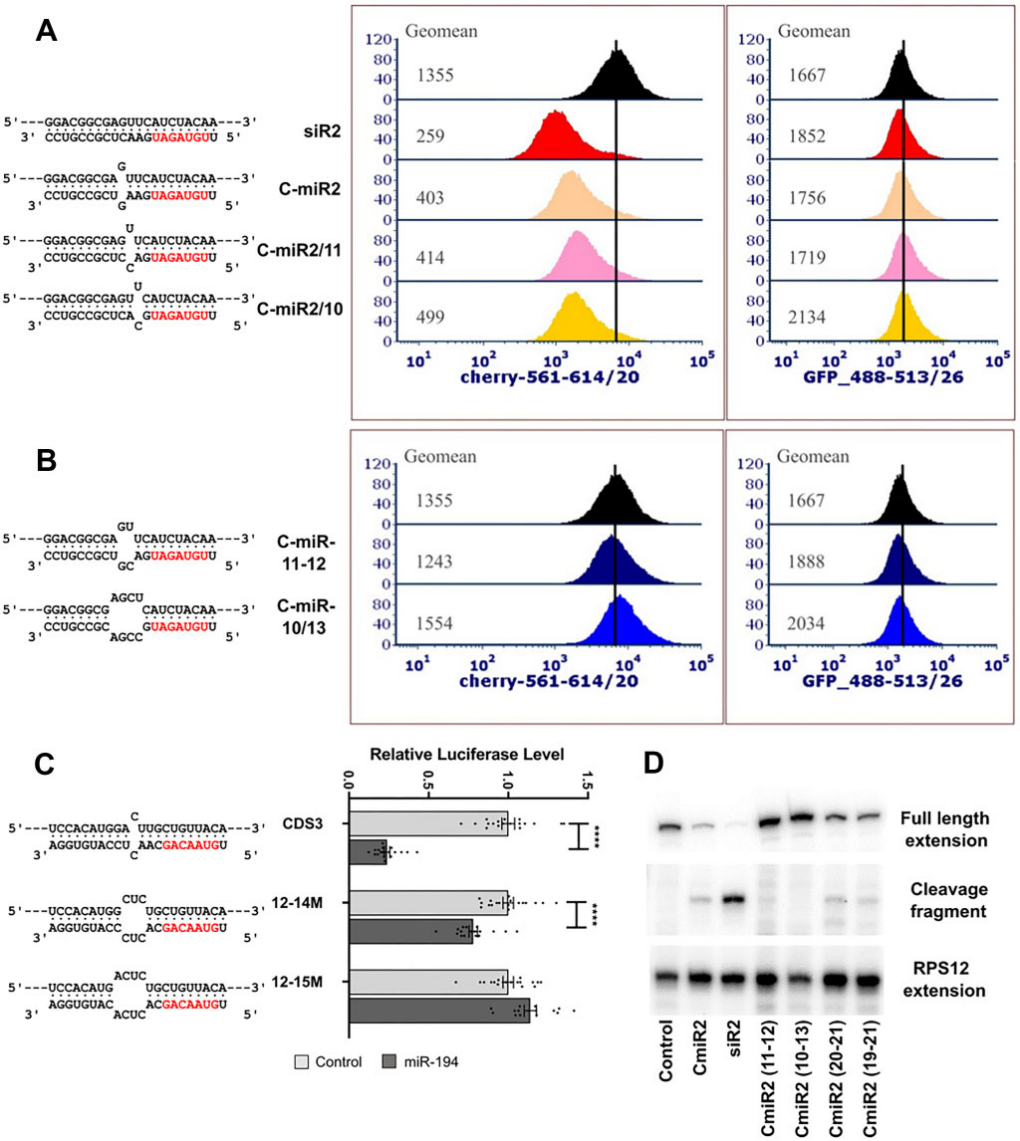
Effects of base pairing disruptions in the centre of the miRNA and primer extension mapping of the cleavage site. (**A**) Effects of single base bulges at miRNA base 12, 11 and 10. (**B**) Effects of larger bulges in the miRNA central region. (**C**) Effects of miR-20a on Renilla luciferase harbouring a CDS site for miR-20a with mismatches in the central region. Data are expressed as mean ± s.e.m., *n* = 18. ****P < 0.0001 as determined by two-tailed Student's *t* test. (**D**) Primer extension assays performed on RNA extracted from MDA-MB-231-eGFP-mCherry cells transfected with the indicated miRNAs (previously featured in Figures 5C, 6A and B). Locations of products consistent with full length mCherry transcript and with miR-directed AGO2-mediated cleavage are indicated. RPS12 primer extension is shown as a loading control.

Although the mismatch in the central region of miRNAs is expected to reduce direct cleavage of target mRNA, we wished to assess the contribution of direct cleavage on mRNA level. The mismatch in the central region of the C-miR2, expected to affect cleavage activity of Ago2 but not miRNA binding (43), reduced the inhibitory effect compared to a fully complementary siRNA (Figure 6A). Single base mismatches at position 12, 11 or 10 of the miRNA (C-miR2, C-miR2/11 and C-miR2/10) all had similar effect, reducing the mCherry level to approximately one third of the level in control cells (Figure 6A). Increasing the size of the bulge in the central region to 2 bases (C-miR2-11-12) severely reduced efficacy while increasing the size to 4 bases (C-miR2-10-13) eliminated activity of the miRNA (Figure 6B). Similar results were found in the luciferase system when the bulge in miR-194 interaction was increased (Figure 6C).

Since these data are consistent with the prime mode of inhibition by the miRNAs being AGO2-mediated direct cleavage, we performed primer extension assays to detect mCherry mRNA that was cleaved at the midpoint of miRNA binding. We found that a primer extension product of the size expected from direct cleavage was present at a level that correlated with the extent of inhibition of mCherry expression, and correlated inversely with the level of uncleaved mRNA, indicated by full length primer extension product (Figure 6D, Supplemental Figure S4). Taken together, these data suggest that in the context of the extensively base-paired interactions that are required for CDS-mediated inhibition, direct cleavage of the target is prominent. Non-cleavage mechanisms such as deadenylation and translational suppression are likely to play lesser roles.

### CDS-mediated targeting of endogenous genes

**Figure 7.**
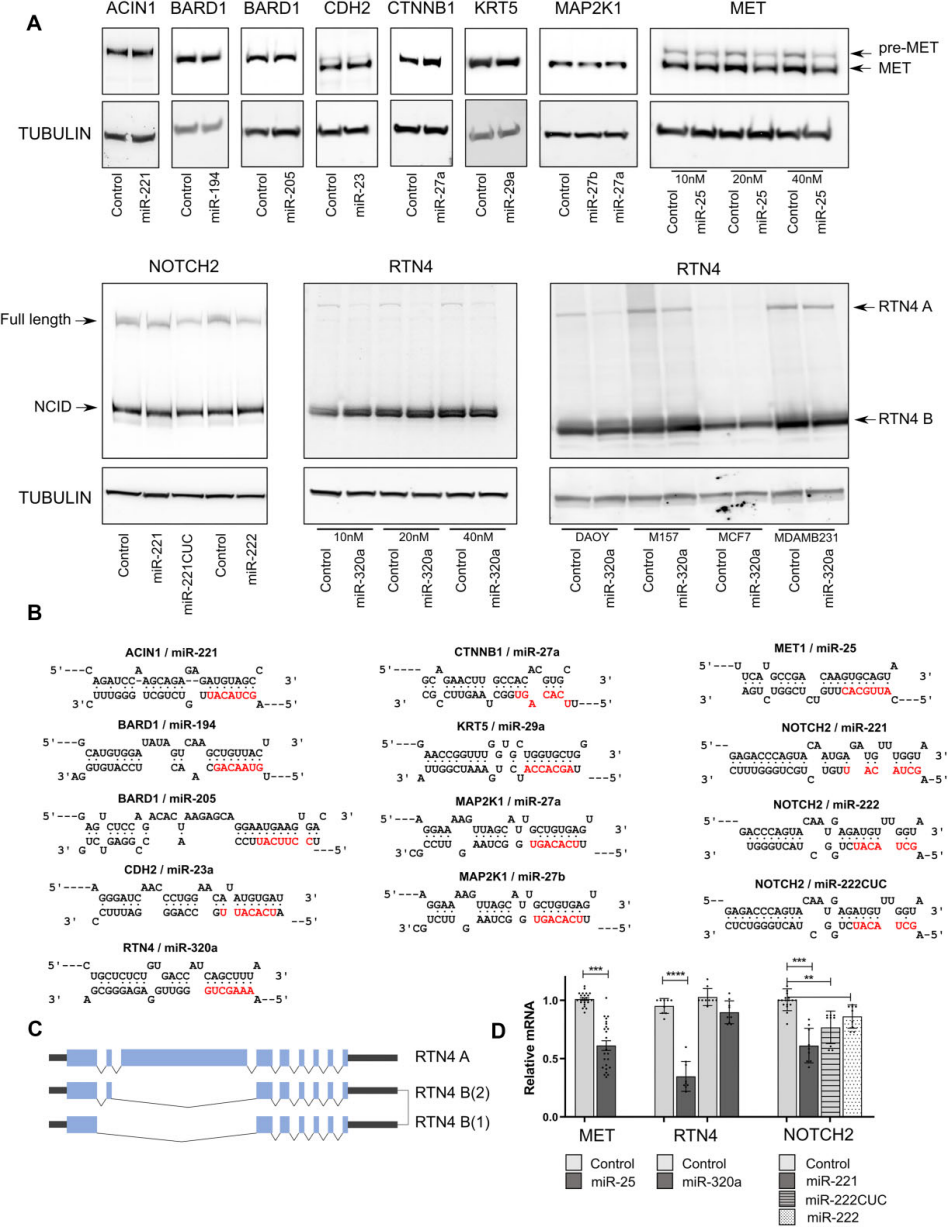
There is minimal repression of endogenous genes by microRNAs targeting coding regions. (**A**) Negative control or a relevant targeting miRNA (10 nM unless otherwise specified) were co-transfected into MDA-MB-231 cells (unless otherwise specified) and western blotting performed to probe expression of the genes indicated. (**B**) Binding models indicating base pairing interactions between the miRNA and the miRNA-response element cloned within the luciferase CDS are shown. The miRNA seed region is shown in red. (**C**) Alternative splicing is responsible for RTN4A and B isoforms. (Pre-Met/Met and NOTCH2/NICD (Notch intracellular domain) are produced via proteolytic processing.) (**D**) Quantitation of mRNA levels normalized to the mean of GAPDH and RPL32 are shown. Data are expressed as mean ± s.e.m. Statistical significance (*P < 0.05, **P < 0.01, ***P < 0.001 and ****P < 0.0001) was determined by two-tailed Student's *t* test.

Our reporter gene experiments indicated that miRNAs targeting CDS regions can be inhibitory so long as there is extensive complementarity and limited bulge size in the central section of the miRNA. To assess whether endogenous miRNAs may target endogenous mRNAs in this manner, we first bioinformatically searched for candidates among human miRNAs and mRNA CDS regions, identifying dozens of candidates with full complementarity within the seed and cleavage region (nucleotides 2–12) and with no more than 2 mismatches throughout the remaining binding interface. Moreover, hundreds of candidate targets are present when 3 mismatches are permitted, with numbers increasing a further 10-fold when the requirement for perfect binding within the three 3′-terminal nucleotides is also removed (Supplemental Table S1). To identify candidates for validation experiments we searched the extensive mouse keratinocyte AGO-CLASH data of Hoefert et al.(36) to identify candidate *in vivo* miRNA-mRNA (CDS) interactions and focused on those with extensive interaction interfaces that are conserved in sequence in humans. In each case that we selected, AGO-CLASH showed binding in the CDS but not the 3′UTR of the respective miRNAs. Based on antibody availability we chose a number of candidates to test by Western blotting after the transfection of cells with the respective targeting miRNAs, but we found little to no repression of the target in most cases. We did observe some repression of MET (by miR-25-3p), NOTCH2 (by miR-221/222-3p) and RTN4 (by miR-320a-3p), but in each of these cases it was primarily a minor isoform of the protein that was affected (Figure 7A, B, Supplemental Figure 4). MET and NOTCH2 are processed into more abundant lower weight forms by protein cleavage, while different isoforms of RTN4 arise from alternative splicing (Figure 7C). Consistent with previous data (Figure 5E), the repression of these minor isoforms was observable at both the protein and RNA level (Figure 7D). These data, along with our extensive reporter approach, indicate miRNAs can exert effects through coding regions, but only if there is extensive base pairing to the target which includes full complementarity with the miRNA seed. We suggest these strict requirements severely limit the impact of CDS sites in all but the most extreme of cases.

## DISCUSSION

The development of high-throughput methodologies to profile miRNA binding has revolutionized the field. Techniques such as AGO HITS-CLIP (40), AGO Par-CLIP (44), AGO-CLASH (41) and AGO CLEAR-CLIP (42) isolate AGO-containing complexes from cells and enable the identification of binding sites *en masse*. AGO ‘bind-and-seq’ assesses all potential binding sites of synthesized oligonucleotides *in vitro* (14). These techniques consistently demonstrate an abundance of miRNA interaction, not only across 3′UTRs where miRNAs are well known to function, but also across coding regions and even introns (30,36,40–42,45,46). This suggests miRNAs may impact genes more frequently than is currently appreciated and/or may target genes that are often ignored by 3′UTR-centric target prediction algorithms.

The mere identification of a binding site however does not necessarily indicate function (47). This is because AGO-pulldown approaches are capable of capturing transient interactions between miRNAs and their targets and even if an interaction is stable, the stoichiometry between miRNA and target might be such that the interaction is of little functional consequence. Even so, the observation remains: miRNAs frequently interact within coding regions and multiple studies report examples where miRNAs binding within coding regions have an impact on gene expression and cell behaviour (27–29,31). For example, it was recently reported that the transfection of miRNAs could increase AGO-occupancy within the CDS and post-transcriptionally downregulate gene expression in an additive manner with increasing numbers of CDS sites (28). The specific importance of one CDS site was also recently demonstrated in the context of granulosa cell tumours, where somatic mutation within the coding region of the tumour suppressor FOXL2, caused FOXL2 haploinsufficiency through the creation of a novel target site for miR-1236 (29).

Of particular interest was a report that miRNAs can bind to coding regions and abort translation in a manner that is dependent upon the 3′ end of the miRNA but not the seed (31). This is of particular interest as it may represent a pool of miRNA targets that have previously gone unrecognized. This is because the seed-less interaction will not be predicted by most algorithms, and the translation-only mechanism will make gene repression invisible in RNA-sequencing and qPCR experiments. Elements of this observation are echoed in other findings. For example, enhancing gene repression by the introduction of non-optimal codons suggests competition exists between RISC and the ribosome (15) and transfection experiments have revealed that sites located in both the 3′UTR and CDS are capable of inhibiting translation (27). Furthermore, RNAi in *C. elegans* functions at the translational level in addition to target cleavage, and generates stalled ribosome-mRNA complexes that are observable in the absence of the factors (SKI and PELOTA) that will otherwise clear them (17). We have sought to clarify whether miRNAs are able to repress genes by binding within coding regions and if so, what are the sequence requirements for this to happen. One would anticipate these requirements to be more extensive in coding regions than 3′UTRs given the necessity of RISC to compete with transiting ribosomes.

By constructing multiple variants of MREs within reporter constructs, we confirmed that miRNAs are capable of repressing gene expression through sites located within coding regions if base pairing is extensive. However, in all examples tested we found targeting to be of the canonical, seed-dependent type. This is not strictly dependent upon perfect complementarity at the 3′ end, but is heavily dependent upon extensive binding across the rest of the interaction interface. This includes binding across the central region, where mutations that bias against direct target cleavage decrease efficiency of repression, whilst mutations sufficient to abrogate cleavage eliminated the suppressive capacity of the miRNA altogether. Accordingly, miRNA-CDS interactions that are repressive cause a reduction in mRNA levels and the production of fragments with termini exactly coincident with the products of miRNA-directed, AGO-mediated cleavage. Although 3′ pairing is not a strict requirement, increasing 3′ mismatches (Figures 3B,E,4A, Supplemental Figures 2B,E) do generally reduce the degree of repression, but this may simply be due to an overall reduction in the strength of target binding as opposed to the special significance of the 3′-terminus. This conclusion is supported by mismatched nucleotides at the 3′ terminus being compensated for by the presence of a longer miRNA : target interaction interface (compare ‘c-miR2 21-23’ with c-miR2 19–21, Figure 5C). Of note, suppression is efficient even when the MRE is situated close to the start codon (‘RL1’ in Figure 1B). This indicates that no-go decay (48) is not associated with repression because the distance of the site from the start codon is insufficient to allow the requisite build-up of stalled ribosomes that leads to transcript turnover.

Whilst we are not able to discern if additional, non-cleavage mechanisms also contribute to repression, our findings demonstrating reduced levels of target transcripts and seed-dependent/3′ terminus-independent binding are in direct contradiction to that previously reported (31), but are supported bythe remarkable consistency of our results between different MRE-reporters, using multiple microRNAs, multiple cell lines and across two entirely separate reporter systems.

In spite of extensive binding requirements, hundreds, if not thousands of candidate interactions between miRNAs and coding regions are either possible (Supplemental Table S1) or experimentally demonstrated (mouse keratinocyte AGO-CLASH data (36), Supplemental Tables S2 and S3). However, we find that if target repression is to be mediated through CDS sites, extensive complementarity along the length of the miRNA : target interface is required. Such interactions are exceedingly rare within CLASH data (Figure 1C) and no trends are observable to suggest that CDS interactions are generally more extensive than 3′UTR sites (Supplemental Figure 5a), nor are predicted CDS binding sites conserved beyond the constraints imposed by the requirements of protein coding (Supplemental Figure 5b). We examined a number of miRNA : mRNA (CDS) candidates identified by mouse CLASH data that retained conserved sequences in humans, but only found modest levels of repression for three out of ten endogenous genes, for which in each case, the effect of the miRNA is only apparent for the lesser expressed isoform (Figure 7). It is possible that for one of these (RTN4), structural RNA differences could explain the differential responsiveness to the targeting miRNA as different exons are present between isoforms, even if the putative MRE is present in all isoforms. However, this cannot be the case for MET and NOTCH2, where isoforms arise from post-translational processing, yet miRNA-mediated suppression could only be detected of the lowly-abundant form. Taken together with our other data, the most likely explanation is that the modest effects of an imperfectly paired miRNA within a CDS are simply swamped by high levels of target expression. If so, CDS targeting may not only require unusually extensive binding, but may also be more specifically relevant for modestly expressed genes. It is noteworthy that a modest degree of repression was observed for miR-221/222-mediated targeting of NOTCH despite seed mis-pairing (there are 2 extra nucleotides in the seed-binding region of NOTCH2 mRNA that otherwise pairs with miR-221 and miR-222CUC). Whether this is the result of repression being mediated via indirect means, or whether such a bulge does not substantially reduce the affinity of binding in this case (which is still sufficient for robust interaction) is unclear.

In agreement with both Zhang et al. and other reports (27–29), our data demonstrate that suppression by MREs located within the CDS can occur, although in contrast to Zhang et al. we find repression requires extensive complementarity that involves the seed region and leads to target cleavage. Although we cannot rule out exceptions to this, we conclude that the extensive binding that is required for efficient MRE-CDS function will likely limit its influence to only a small number of modestly expressed genes.

## Supplementary Material

gkad645_Supplemental_Files

## Data Availability

All data is freely available on request to the corresponding author. Full sequences to small RNAs, primers and control RNAs are provided in the article. Full length gels are included as supplementary figures.
