# Supplementary material for: On the rules of engagement for microRNAs targeting protein coding regions
Source: Nucleic Acids Res. 2023 Jul 31;51(18):9938–51. doi: 10.1093/nar/gkad645 (PMC10570018; doi:10.1093/nar/gkad645)
Supplement: gkad645_Supplemental_Files [file gkad645_supplemental_files.zip › Supplemental Figures and Tables_Sapkota et al_resubmission.pdf]

SUPPLEMENTAL DATA

On the rules of engagement for microRNAs targeting protein coding regions

Sunil Sapkota[1], Katherine A Pillman[1,2], B Kate Dredge[1], Dawei Liu[1], Julie M Bracken[1], Saba Ataei Kachooei[1], Bradley Chereda[1], Philip A Gregory[1,3], Cameron P Bracken[1,3,4]* and Gregory J Goodall[1,3,4]*

SUPPLEMENTAL FIGURE AND TABLE LEGENDS

Supplemental Figure 1. Predicted local secondary structure in regions of Renilla luciferase targeted by the artificial miRNAs

The local folding predicted by Mfold (49) is shown, with the bases targeted by the artificial miRNAs shown in blue.

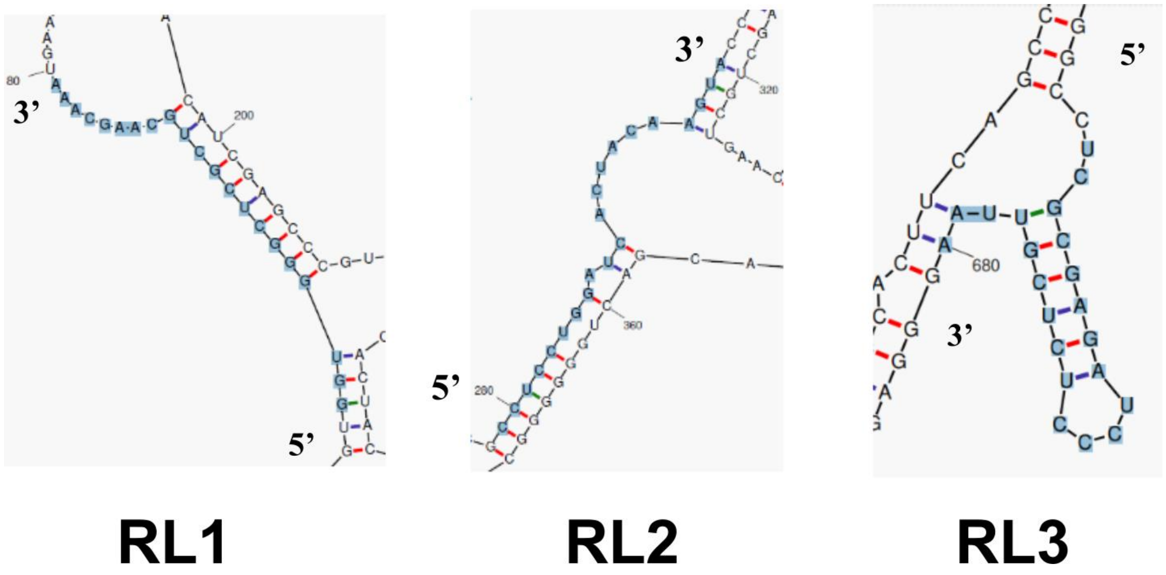

Supplemental Figure 2. Base-pairing interactions required in the CDS or 3'UTR for miR-200b and miR-194 to effectively target the CDS of a luciferase reporter gene.

(**A,D**) Binding models indicating base pairing interactions between the miRNA the miRNA-response element cloned within the luciferase CDS. The miRNA seed region is shown in red. (**B,E**) Results of Renilla Luciferase reporter assays after co-transfection of **(B)** miR-200b or **(E)** miR-194 mimic in MDA-MB-231 cells. **(C)** Results of Renilla Luciferase reporter assays after co-transfection of miR-200b inhibitor in MCF7 cells. All quantitative data are based on three biological replicates, with each experiment containing 6 technical replicates. Data are expressed as mean ± s.e.m. Statistical significance (**P* < 0.05, ***P* < 0.01, ****P* < 0.001 and *****P* < 0.0001) was determined by two-tailed Student's *t* test.

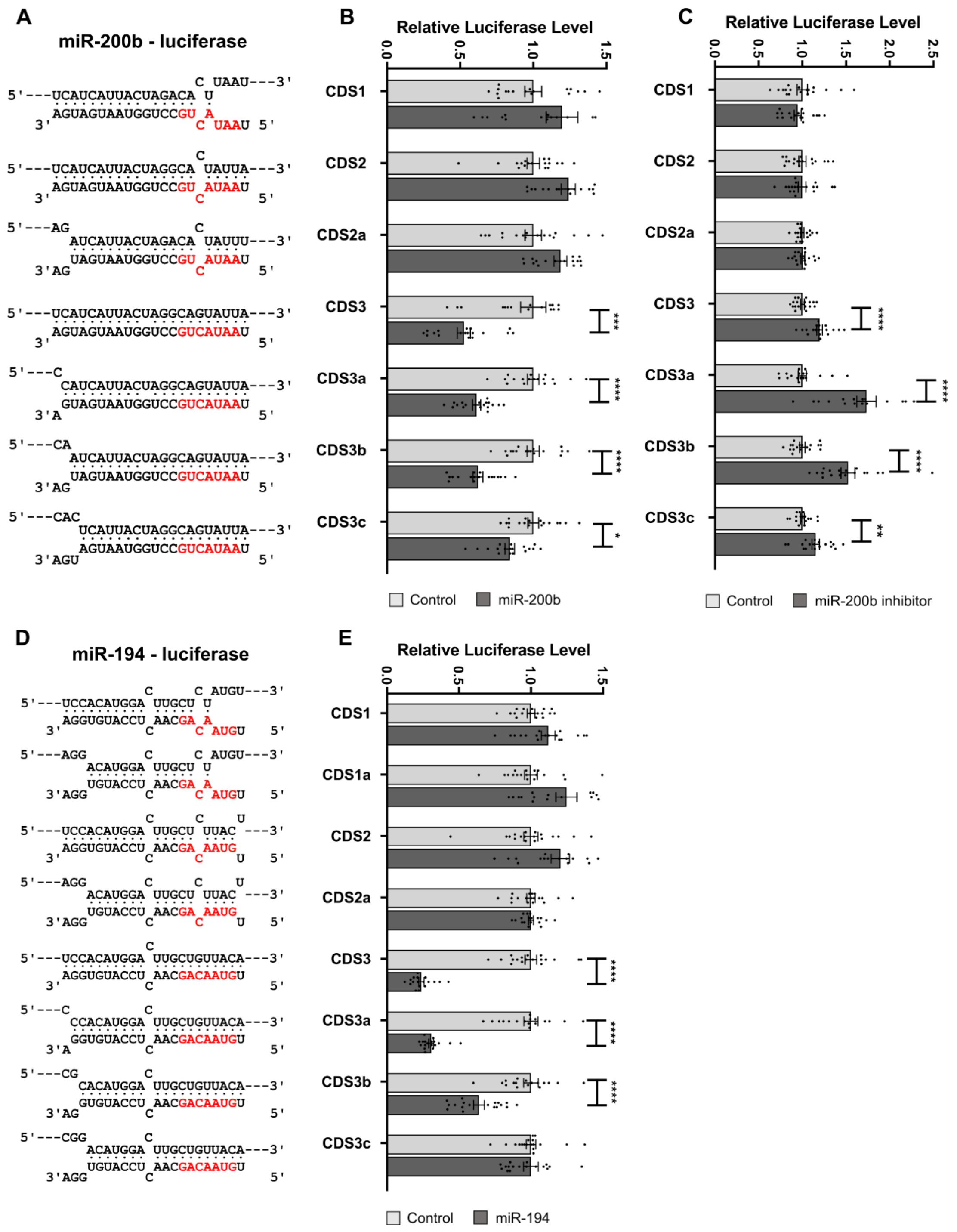

Supplemental Figure 3. miR-20a does not suppress a reporter construct derived from the putative CDS-miR-20a binding site of DAPK3.

A) Results of Renilla Luciferase reporter assays after co-transfection of either a control RNA or mimics of miR-20a or miR-194. Reporter constructs relevant to either miR-20a or miR-194 are the same as those shown in Figure 2A, 4A (miR-20a), Figure 4B (DAPK3) or Supplemental Figure 2D (miR-194). **B**) A let-7b-responsive reporter (perfect let-7b target site) is suppressed by let-7b expression. All transfections were performed in HeLa cells. Data are expressed as mean ± s.e.m. Statistical significance (**P* < 0.05, ***P* < 0.01, ****P* < 0.001 and *****P* < 0.0001) was determined by two-tailed Student's *t* test.

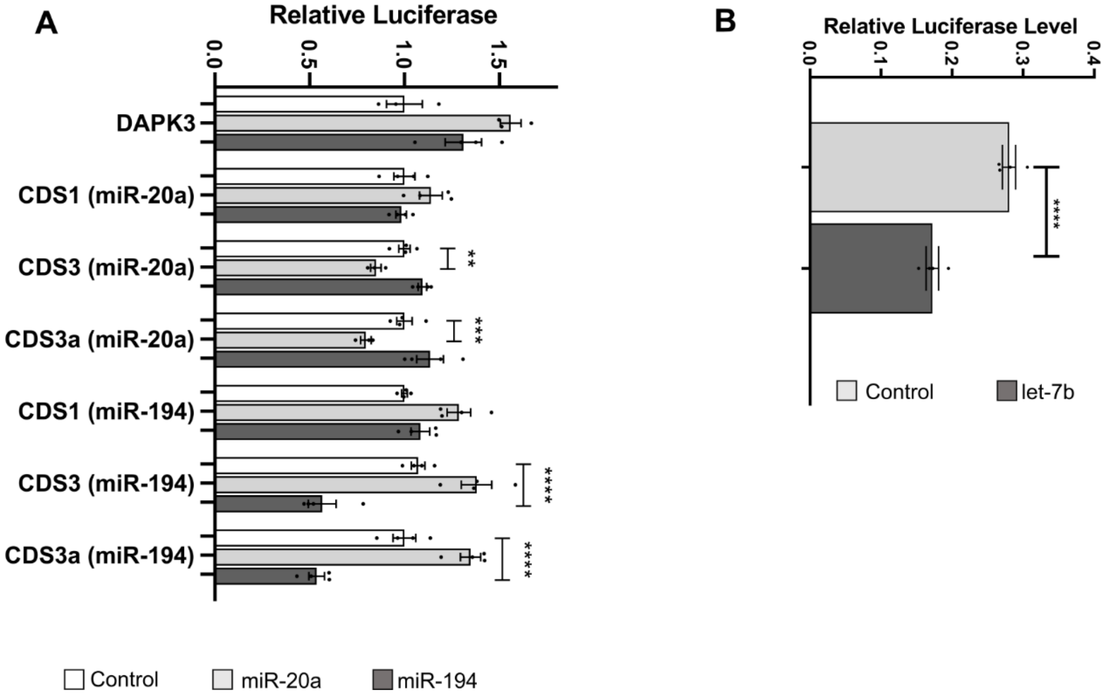

Supplemental Figure 4. Full images of publication figures

(A), Locations of cleavage sites and expected fragment sizes resulting from pseudomiR-directed cleavage of the mCherry transcript. **(B-C)** Full gel images of **(B)**, mCherry primer extension qRT-PCR (from Fig. 6D) and **(C)** immunoblots of proteins (from Fig. 7). Blots of NOTCH2 and RTN4 are not included as those provided in Figure 7 are full size.

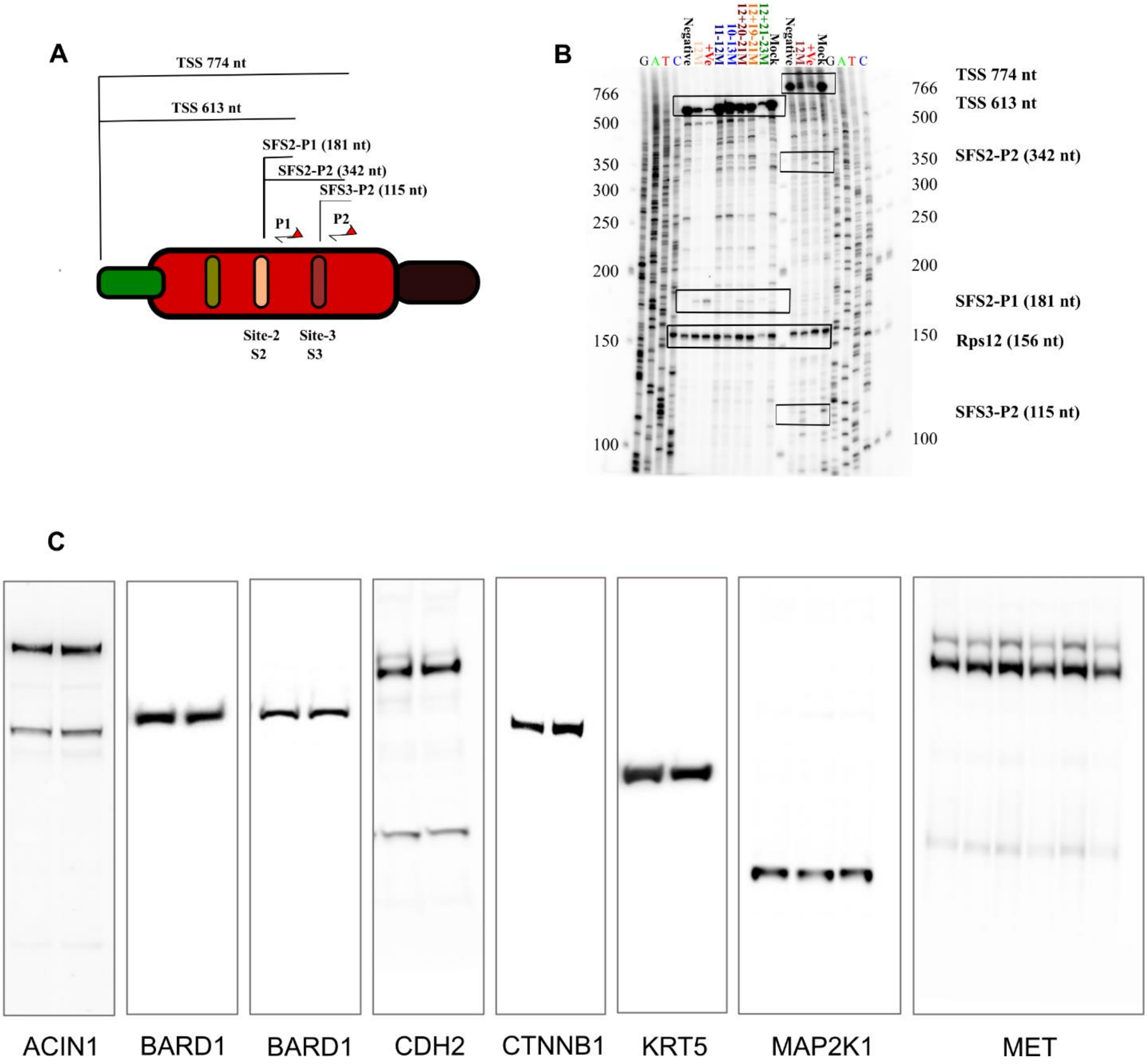

Supplemental Figure 5. miRNA binding sites within the CDS resemble 3'UTR binding sites and are not additionally conserved.

(**A**) Binding profiles of the 30 most expressed miRNAs from mouse AGO-CLASH, divided into CDS and 3'UTR located sites. (**B**) Mean 100 vertebrate basewise conservation by PhyloP scores are calculated from predicted CDS target sites of the same 30 miRNAs in (A). Conservation scores are compared between the 8nt seed-pairing site and the mean of conservation scores for the 50 exonic nucleotides 5' and 3' to the seed site.

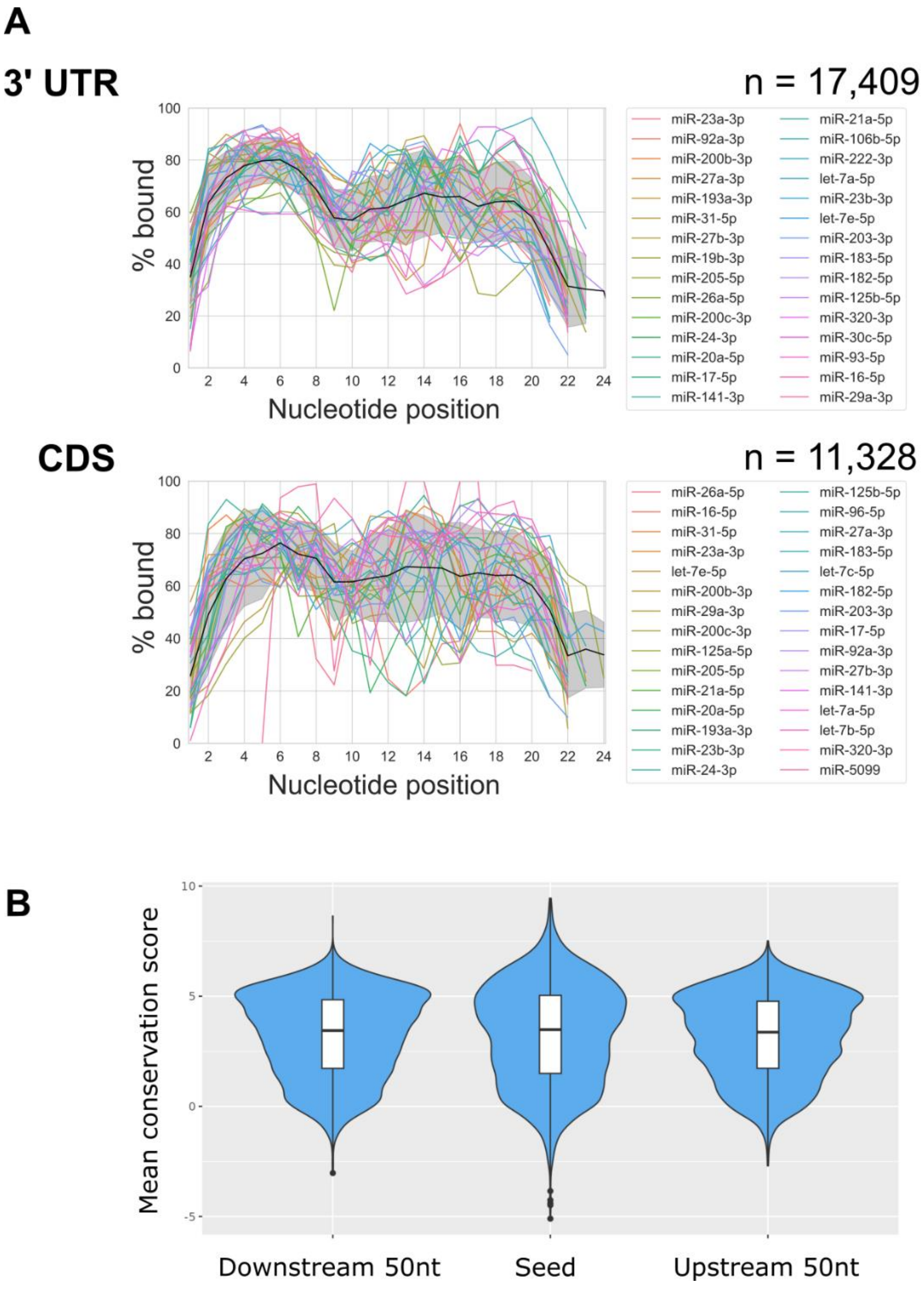

Supplementary Table S1: Extensive interactions between microRNAs and coding regions are predicted

Numbers of potential interactions between microRNAs and either 3'UTR or coding region sequences are indicated based upon the stated base pairing requirements. Genomic sequences were extracted from Ensembl Biomart. 560 microRNAs were considered that were annotated as either high confidence with >10 reads per million in miRBase v22.1, or low confidence but with >1000 reads per million.

Supplementary Table S2: Interactions between microRNAs and their targets within 3'UTRs in AGO-CLASH data

All the interactions between miRNAs and mRNA transcripts within 3'UTRs identified in the mouse AGO-CLEAR-CLIP data from Hoefert et al. (36) are shown. Interactions are ordered by deltaG, with predicted base pairing indicated within the dot:bracket column.

Supplementary Data Table S3: Interactions between microRNAs and their targets within the CDS in AGO-CLASH data

All the interactions between miRNAs and mRNA transcripts within coding regions identified in mouse AGO-CLEAR-CLIP data from Hoefert et al. (36) are shown. Interactions are ordered by deltaG, with predicted base pairing indicated within the dot:bracket column.

Supplementary Data Table S4: Reagents

G-blocks and single oligonucleotides used for cloning, and sequences (or catalog details) for miRNA mimics, miRNA inhibitors, transfected pseudomiRs and qRT-PCR primers are indicated.
